# Prevalence, contributing factors, and interventions to reduce medication errors in outpatient and ambulatory settings: a systematic review

**DOI:** 10.1007/s11096-023-01626-5

**Published:** 2023-09-08

**Authors:** Lina Naseralallah, Derek Stewart, Malcom Price, Vibhu Paudyal

**Affiliations:** 1 School of Pharmacy, College of Medical and Dental Science, Institute of Clinical Sciences, Sir Robert Aitken Institute for Medical Research, University of Birmingham, Edgbaston, Birmingham, B15 2TT UK; 2 Clinical Pharmacy and Practice Department, College of Pharmacy, QU Health, Qatar University, Doha, Qatar; 3 Institute of Applied Health Research, University of Birmingham, Birmingham, UK; 4 NIHR Birmingham Biomedical Research Centre, University Hospitals Birmingham NHS Foundation Trust, Birmingham, UK

**Keywords:** Ambulatory, Contributory factors, Medication errors, Outpatient, Systematic review

## Abstract

**Background:**

Medication errors are common events that compromise patient safety. Outpatient and ambulatory settings enhance access to healthcare which has been linked to favorable outcomes. While medication errors have been extensively researched in inpatient settings, there is dearth of literature from outpatient settings.

**Aim:**

To synthesize the peer-reviewed literature on the prevalence, nature, contributory factors, and interventions to minimize medication errors in outpatient and ambulatory settings.

**Method:**

A systematic review was conducted using Medline, Embase, CINAHL, and Google Scholar which were searched from 2011 to November 2021. Quality assessment was conducted using the quality assessment checklist for prevalence studies tool. Data related to contributory factors were synthesized according to Reason’s accident causation model.

**Results:**

Twenty-four articles were included in the review. Medication errors were common in outpatient and ambulatory settings (23–92% of prescribed drugs). Prescribing errors were the most common type of errors reported (up to 91% of the prescribed drugs, high variations in the data), with dosing errors being most prevalent (up to 41% of the prescribed drugs). Latent conditions, largely due to inadequate knowledge, were common contributory factors followed by active failures. The seven studies that discussed interventions were of poor quality and none used a randomized design.

**Conclusion:**

Medication errors (particularly prescribing errors and dosing errors) in outpatient settings are prevalent, although reported prevalence range is wide. Future research should be informed by behavioral theories and should use high quality designs. These interventions should encompass system-level strategies, multidisciplinary collaborations, effective integration of pharmacists, health information technology, and educational programs.

**Supplementary Information:**

The online version contains supplementary material available at 10.1007/s11096-023-01626-5.

## Impact statements

- Medication errors are common in outpatient and ambulatory settings, with prescribing errors and dosing errors being the most prevalent.
- Latent conditions, including inadequate training or knowledge, were more common followed by active failures. Mistakes and violations were the most frequent contributory factors related to active failures.
- There is a need for the development of theory-based multifactorial interventions to minimize medication errors in outpatient and ambulatory settings.
- Randomized controlled trials are needed to develop and evaluate the long-term outcomes of complex interventions in these settings.

## Introduction

Medication errors represent a serious public health problem posing a threat to patient safety [1]. According to the World Health Organization (WHO), medication errors injure 1.3 million people annually and cause one death daily in the US [2]. Additionally, WHO estimated the global impact of medication errors to be approximately $42 billion annually [3]. Hence, improving medication safety has been declared by the WHO as the third global patient safety challenge [4]. A myriad of potential interventions has been proposed to mitigate medication errors, including pharmacist-led interventions, educational interventions, technology-driven interventions, and multidisciplinary team implementation [5–10].

Outpatient and ambulatory settings can be defined as medical settings that provide general or specialized services that do not warrant hospital admission [11, 12]. These settings minimize admission-related complications and costs while maintaining the same quality of care to inpatient setting [13–15]. Additionally, high-quality outpatient services increase patient satisfaction, promote prophylactic healthcare, provide sustainable management of chronic diseases, reduce unplanned doctor visits and hospitalization, and reduce mortality [16–22]. Therefore, establishment of these settings has been prioritized by healthcare systems in recent years alongside integrated models with primary care services [15, 23]. The introduction of technological innovations has also permitted diagnostic and interventional procedures to be performed without hospitalization thereby expanding the role of outpatient and ambulatory settings [13–15].

Recent studies from the US and the UK highlight that the prevalence of medication errors in outpatient and ambulatory sectors is high [1, 24–26]. For instance, the National Health Service (NHS)-England reported that four of every ten errors take place in outpatient and ambulatory settings [1]. Additionally, around three quarters of the 66 million clinically important errors that occur annually were also in these settings [1].

Whilst multiple systematic reviews have explored the rates, nature, and contributory factors to medication errors in diverse inpatient settings [6, 27–30], synthesis of evidence from outpatient and ambulatory settings is lacking [31]. There is a rising demand for healthcare policy to manage patients in these settings to minimize healthcare costs and resources, and enhance patient access to services [23]. Findings from such synthesis could enable policy makers to estimate the extent of the problem; understand the nature of these errors; and design effective interventions targeting the identified contributory factors.

### Aim

The aim of this systematic review was to synthesize the peer-reviewed literature on the prevalence, nature, contributory factors, and interventions to minimize medication errors in adult population visiting outpatient and ambulatory settings.

## Method

The reporting of this systematic review follows the recommendations provided by the Preferred Reporting Items for Systematic Reviews and Meta-Analyses (PRISMA) guidelines [32]. The research protocol was registered with the International Prospective Register of Systematic Reviews (PROSPERO)-CRD42021291006 [33].

### Eligibility criteria

Articles were considered for inclusion if they met the following criteria: (1) reported prevalence or contributary factors; (2) conducted in hospital-based outpatient clinics or ambulatory care facilities; (3) adult patients (≥ 18-years); (4) English language; (5) published from 2011 onwards. Studies that included medication errors taking place in both inpatient and outpatient settings were only included if outpatient setting data were presented separately to the inpatient data. For the purpose of this study, we adopted the National Coordinating Council for Medication Error Reporting and Prevention (NCCMERP) definition of medication errors “any preventable event that may cause or lead to inappropriate medication use or patient harm while the medication is in the control of the health care professional, patient, or consumer” [34]. We also captured the definitions of medication errors used by individual studies.

Papers published prior to 2011 were excluded as advances in healthcare in recent years were deemed to outdate prevalence data from older reports [13, 14]. Studies focusing on adverse drug events (i.e. harm experienced by a patient as a result of exposure to a medication; adverse drug events encompasses a wide range of incidents such as adverse drug reactions and medication errors) with lack of clear relevance to medication errors were excluded [35]. Additionally, studies focusing on pediatric patients were excluded due to the known factors in relation to development processes making this population more prone to experiencing medication errors [6]. Editorials, commentaries, reviews, case-studies, and conference abstracts were also excluded.

### Data sources and search strategy

The search was undertaken in the following electronic bibliographic databases and search engines from 2011 until November 2021: Medline, Embase, and Cumulative Index of Nursing and Allied Health Literature (CINAHL). Google Scholar (first 500 records) was screened manually for additional records by one reviewer and potentially eligible records were imported to EndNote to check if they were duplicates. Reference lists of included articles were reviewed to locate potentially relevant studies not identified through database searching.

Search terms were: (medication error OR ((medication* OR transcrib* OR prescrib* OR dispens* OR administ*) adj3 (incident* OR mistake* OR error*)) AND (outpatient clinics, hospital OR ambulatory care OR ambulatory care facilities OR outpatients OR ((ambulatory OR outpatient*) adj3 (care* OR healthcare* OR clinic* OR service* OR department* OR center* OR facilit*))).

### Study selection

Database hits and identified references were transferred to EndNote 20® (2021 Clarivate) to remove duplicates. The remaining articles were imported into Rayyan Qatar Computing Research Institute (QCRI) software [36], for title and abstract screening followed by full text screening using Microsoft Excel. Screening was conducted by two independent investigators (LN, VP or DS), with other research team members involved in cases of disagreement.

### Data extraction

Data extraction was conducted by one reviewer (LN) and independently verified by a second (VP). A pre-piloted data extraction form was used to extract the following: author, year of publication, country, setting, aim, duration, study design, participant sampling and recruitment, error prevalence (all relevant data), nature of errors, error severity, contributory factors, and intervention characteristics and outcomes (if any).

### Risk of bias

Quality assessment was conducted by one reviewer (LN) and independently verified by a second (VP). The quality assessment checklist for prevalence studies was used [37]. This validated tool was developed for the purpose of examining the risk of bias in prevalence studies and is suggested to be user friendly offering high interrater agreement [37]. This is a 10-question tool, with the last item being an overall risk of bias score. Studies were considered of low risk if the final score was 0–3 points, moderate if the score was 4–6, and high risk if the total score was 7–9.

### Data synthesis and statistical analysis

A narrative approach to data synthesis was employed for data related to classification, nature, and contributory factors. Narrative synthesis can be defined as “an approach to the systematic review and synthesis of findings from multiple studies that relies primarily on the use of words and texts to summarize and explain the findings of the synthesis” [38]. Findings are presented in textual form and summary tables.

Data related to contributory factors were synthesized using Reason’s Accident Causation Model [39]. This model was proposed in 1997 and it was one of the early models that recognized the systemic environment influence on accident phenomenon. By doing so, the system focuses on no-blame culture that aims to understand the multiple factors occurring at different levels of the system and contributing to an incident. This model provides an insight into possible methods of preventing accidents by eliminating contributory factors while previous models have limited usability in term of their prevention [40, 41]. This framework classifies contributory factors into two broad categories of active failure (person approach: unsafe acts committed by frontliners) and latent conditions (system approach: system failures attributed to top level management decisions). Active failures were grouped into slips (error of attention), lapses (error of memory), mistakes (decision-making), and violations (intentional rule breaking) [42]. The contributory factors reported in the included studies were examined to classify them according to the model categories.

Although meta-analysis was planned, it was judged inappropriate due to the high levels of clinical and methodological heterogeneity. Statistical analyses without pooling were carried out by a statistician (MP) with Stata version 16 Statistical Software (StataCorp. 2019. Stata Statistical Software: Release 16. College Station, TX: StataCorp LLC). For proportions, the 95% confidence intervals were calculated using exact Binomial methods. For rates, the 95% confidence intervals were calculated assuming a Poisson distribution for events and normality assumed on the natural log-rate scale.

## Results

### Study selection

**Fig. 1:**
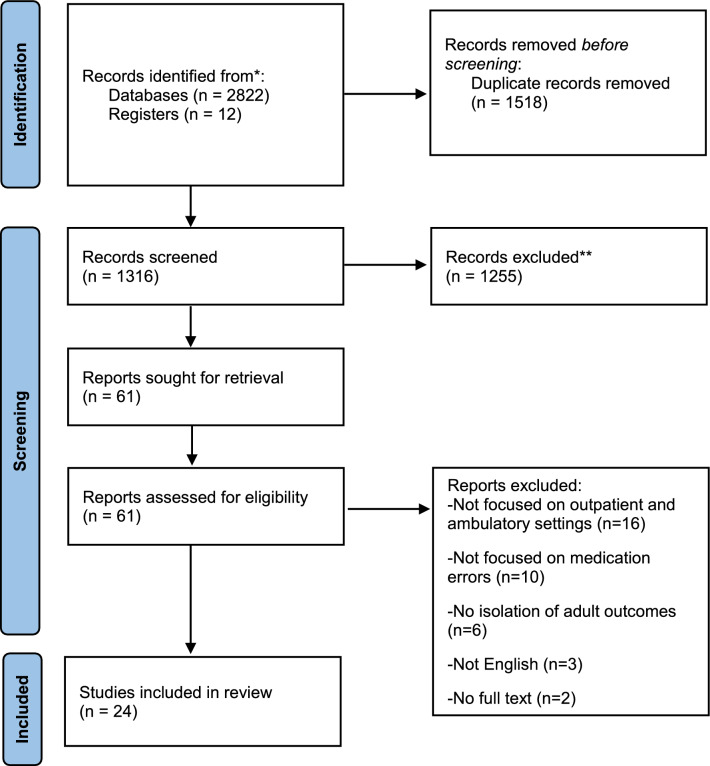
PRISMA flow diagram of the study selection process

Figure 1 presents a PRISMA chart of results of our search strategy and studies included in this synthesis. A total of 1316 unique titles were identified from the database search and reference lists screening. Of these, 61 were reviewed in full text, and 24 fulfilled the inclusion criteria. The reasons for excluding articles at the full-text screening stage are presented in the Electronic Supplementary 1, Table 1.

**Table 1:** Study characteristics

Author	Country, setting	Duration	Study design	Participant sampling and recruitment, total number of participants	Total number of observations (denominator)	Population/data characteristics	Study outcomes	Medication most frequently attributed with ME
Abramson E et al [43]	USA, ambulatory care centers	15 months	Non-randomized cross-sectional study	All prescriptions were evaluated, ensuring that at least 75 prescriptions on 25 per provider patients were obtained and extending data collection if necessary. Prescription review was limited to three randomly selected prescriptions per patient to minimize clustering of errors	5955 patients (9385 prescriptions)	New York: Mean age: 54 years (SD 17), Female: 2388 (63%) Massachusetts: Mean age: 51 years (SD 18), Female: 1324 (62%)	PE Types of PE Intervention Contributory factors	Antibiotics: 1516 (16.4%) Dyslipidemia drugs: 530 (5.7%) Narcotics: 500 (5.4%)
Abramson E et al [44]	USA, outpatient clinic	3 months	Mixed methods cross-sectional case study	Electronic prescriptions were extracted from the electronic database for a 2-week period	920 patients (1905 prescriptions)	Mean age: 57 years (SD 16), Female: 632 (69.2%)	PE Types of PE Contributory factors	Vitamins: 9 (12.7%) Inhaled bronchodilators: 5 (7.0%) Antihistamines: 4 (5.6%)
Bell S et al. [45]	USA, ambulatory care practices	5 months	Survey study	NR	22,889 patients	Mean age: 55.16 years (SD 15.96), Female: 14 447 (63.1%)	Overall serious ME Contributory factors	NR
Bicket M et al. [46]	USA, outpatient departments at a tertiary medical center	15 days	Retrospective study	All opioid medication prescriptions received and processed by one outpatient pharmacy for 15 consecutive days	451 patients (510 prescriptions)	Mean age: 47.5 years (SD 17.4), Handwritten prescriptions: 234 (47%)	Overall ME Contributory factors	Study focused on opioids only Tablet form: 92%
Dempsey J et al. [47]	USA, heart failure clinic, or Ambulatory Cardiac Triage, Intervention, and Education unit	5 months	Cross-sectional study	Consecutive visits to the heart failure subspecialty clinic, or ACTIVE unit, that included pharmacist consultation	60 patients	Mean age: 69, Male: 36 (60%), Mean number of medications: 14	PE Types of PE Intervention Contributory factors	Study focused on heart failure only NR
Howard M et al.[48]	USA, family medicine, internal medicine, and geriatrics clinics	6 months	Retrospective chart review	Patients were identified by the institution’s electronic health record by having an active DOACs on their medication list for the study duration	167 patients (167 drugs/prescriptions)	Mean age: 69.7 years (SD 15.5). Female: 68 (40.7%)	Dosing errors Contributory factors	Study focused on DOACs only
Prasad D et al.[49]	India, outpatient general medicine department	6 months	Cross sectional, interventional study	All patients who visited the clinic and met the inclusion criteria were collected randomly at the dispensing area in the pharmacy	544 patients (544 prescriptions, 1768 drugs)	Age 41–50: 68 (22%), Female: 169 (56%), Diagnosis not mentioned: 73 (24.1%)	Overall ME ME according to the use process Types of PE Severity Contributory factors	Vitamins: 386 (21.8%) Gastrointestinal drugs: 370 (20.9%) NSAIDs: 307 (17.4)
Priya K et al. [50]	India, outpatients in a hospital	12 months	Prospective study	NR	23,750 drugs	NR	PE Types of PE Severity Intervention Contributory factors	NR
Shakuntala B et al. [51]	India, outpatient ophthalmology department at a hospital	4 months	Prospective, observational, and cross-sectional study	Adult patients who registered newly and visiting ophthalmology outpatient department for curable complaints were included	900 patients (900 prescriptions, 1400 antibiotic)	Age 31–60: 423 (47%), Female: 378 (42%), Mean drugs/prescription: 2.62	PE Types of PE	Study focused on antibiotics only Fluoroquinolones: 1218 (87%) Eye drops: 69%
Thakur et al. [52]	India, medicine department in a hospital	5 months	Prospective cohort study	NR	100 patients	NR	Overall ME	NR
Al-Khani S et al. [53]	Saudi Arabia, ambulatory care setting	21 months	Retrospective study	All prescribing errors reported during the duration of the study were included	NR	NR	PE Types of PE Contributory factors	NR
Assiri G et al. [54]	Saudi Arabia, family medicine clinics	18 months	Retrospective cohort study	Several ambulatory care centers were contacted for fieldwork selection. Family Medicine clinics in two hospitals were selected. A random sample of patients visiting the clinics was generated	2000 patients	Mean age: 49.9 years, Female: 1302 (65.1%), Polypharmacy: 1,115 (55.8%)	Overall clinically important ME ME according to the use process Types of PE Contributory factors	NR
Carollo J et al.[55]	Brazil, outpatient chemotherapy unit of a teaching hospital	3 months	Cross-sectional and descriptive study	The calculation of minimal sample to develop the study was based on 12,778 health care procedures done in 2015. Recruitment not mentioned	1403 patients [1, 403 healthcare procedures]	Mean age: 57.6 years (SD 15.2), Female: 819 (58.4%)	Overall ME ME according to the use process Types of PE Severity Contributory factors	Study focused on chemotherapy only IV route of administration: 680 (48.5%)
Duarte et al. [56]	Brazil, outpatient oncology and chemotherapy clinic at a hospital	6 months	Prospective observational study	Prescriptions for all patients who were treated with chemotherapy during the study period were delivered daily to the chemotherapy pharmacy service by the nursing staff and/or clinical staff	780 patients (3526 prescriptions)	Mean age: 60.6 years (SD 13.2), Female: 262 (33.64%)	PE Types of PE Severity Intervention Contributory factors	Study focused on chemotherapy only
Al Khawaldeh T et al. [57]	Jordan, hematology and oncology outpatient departments at hospitals	6 weeks	Prospective cross-sectional study	NR	334 drugs administered/prescriptions	NR	Administration errors Contributory factors	Study focused on IV chemotherapy only
Belaiche S et al. [58]	France, outpatient nephrology clinics at a university hospital	15 months	Retrospective study	All patients seen by the clinical pharmacist during the study duration but analyzed the data of only those patients seen more than twice, so as to observe any benefit from the introduction of pharmaceutical care	42 patients (350 pharmaceutical consultations, 287 drugs)	Mean age: 64.9 years (SD 2.2), Female: 21 (50%), Stage 4 CKD: 17 (40.5%), Stage 3 CKD: 16 (38.1%), Mean number of drugs: 8.6 (SD 0.6)	Overall ME ME according to the use process Types of PE Intervention Contributory factors	Cardiovascular drugs: 95 (33.1%) Gastrointestinal drugs: 82 (28.6%) Blood and blood derivatives: 62 (21.6%)
Hernández S et al. [59]	Puerto Rico, 330 ambulatory health care centers	4 years	Observational retrospective cohort study	The study sample was selected by convenience in a nonrandomized selection from event reports completed in those years	2218 patients	Mean age: 73.4 (SD 7.4), Female: 112 (65.9%), Mean number of medications: 6.8 (SD 3.9)	Overall ME ME according to the use process Severity Contributory factors	Anticoagulants: *p*-value < 0.001
Kim G et al. [60]	South Korea, 43 medical institutions with hemodialysis facility	3 months	Cross-sectional study	10% of centers with hemodialysis were selected by systematic sampling. Nurses in filled out the questionnaire using medical records and hemodialysis data to recruit all patients who met the inclusion criteria	828 patients (1097 drugs)	Age 18–49: 230 (27.8%), age 50–59: 231 (27.9%), male: 497 (60%), GFR < 10 mL/min/1.73 m2: 785 (94.8%), duration of hemodialysis 1–5 years: 376 (45.4%)	Dosing errors Contributory factors	Study focused on 85 drugs in three classes: antihypertensives, antihyperglycemics and dyslipidemia drugs
Lee P et al. [61]	Singapore, kidney transplant ambulatory clinic	19 months	Prospective observational study	All ME and medication discrepancies documented at the clinic during the study duration were retrieved from the system for analysis	1271 patients (3581 prescriptions)	NR	PE Types of PE Intervention Contributory factors	Immunosuppressive drugs: 25.3% Anti-infectives: 14.1% Antihypertensive drugs: 12.0%
Niriayo Y et al.[62]	Ethiopia, ambulatory care heart failure clinic at a teaching hospital	12 months	Prospective observational study	Patients were recruited during their appointment for medication refilling. A sample of 355 was calculated using a single population proportion formula assuming 50% proportion of ME	340 patients (1389 drugs)	Mean age: 50.5 years (SD 15.6), Female: 171 (50.3%), Mean comorbidities per patient: 1.9 (SD 0.9), New York Heart Association (NYHA) classes III: 165 (48.5%)	PE Types of PE Contributory factors	Study focused on heart failure only Beta-blockers: 34.4% Angiotensin-converting-enzyme inhibitors (ACEIs): 24.8% Dyslipidemia drugs: 16.5%
Ojeh V et al. [63]	Nigeria, outpatient HIV clinic at a teaching hospital	8 months	Prospective descriptive study	All HIV infected adults that presented at the pharmacy with prescription for routine ART pick up or initiation during the study duration	9339 patients [42, 416 prescriptions]	Mean age: 41 years (SD 10), Female: 6,817 (73%)	PE Types of PE Intervention Contributory factors	Study focused on antiretroviral drugs only
Rouhani M et al. [64]	Iran, outpatient cancer centers	6 months	Prospective, cross-sectional interventional study	All standard forms were collected, and ME and possible side effects were evaluated	84 patients (217 cycles, 385 drugs)	Breast cancer patients. Mean age: 46.17 years (SD 9.5). Female: 81 (96.4%)	Overall ME ME according to the use process Types of PE Contributory factors	NR
Shaikh A et al. [65]	Pakistan, outpatient departments in hospitals and primary healthcare facilities	NR	Retrospective study	NR	479 prescriptions	Missing diagnosis: 402 (84%) prescriptions	PE Types of PE Contributory factors	Study focused on NSAID only
Shrestha R et al. [66]	Nepal, outpatient departments at a hospital	2 months	Retrospective, cross-sectional, and quantitative study	The sample was selected using stratified (according to department) random sampling by dividing the sample number based on the prescription number of each department	770 prescriptions, 2448 drugs	Mean drugs/prescription: 3.2	PE Types of PE Severity Contributory factors	NR

*ME* Medication errors; *PE* Prescribing errors; *SD* Standard deviation; *NR* Not reported; *CKD* Chronic kidney disease; *NSAID* Non-steroidal anti-inflammatory drugs; *ADE* Adverse drug events; poADE: Potential ADEs; *DOAC* Direct oral anticoagulants; *DTP* Drug therapy problem; *ART* Antiretroviral therapy

### Characteristics of included studies

The characteristics of included studies are presented in Table 1. Of the 24 included studies, six were conducted in the US [43–48], four in India [49–52], and two each in Saudi Arabia [53, 54] and Brazil [53–56]. One study was conducted in each of Jordan [57], France [58], Puerto Rico [59], South Korea [60], Singapore [61], Ethiopia [62], Nigeria [63], Iran [64], Pakistan [65], and Nepal [66]. Thirteen studies were prospective or retrospective cohort studies [46, 48, 50, 52–54, 56, 58, 59, 61–63, 65] and eleven were cross-sectional studies [43–45, 47, 49, 51, 55, 57, 60, 64, 66]. Follow-up duration ranged from 15 days [46] to 4 years [59].

Most studies (*n* = 18) recruited participants from outpatient clinics [44, 46–52, 54–58, 61–66], while six were from ambulatory centers [43, 45, 53, 59–61]. Although most studies (*n* = 13) did not focus on a particular medical subspeciality, eleven focused exclusively on a single pharmacological class or disease state (Table 1). Among studies that did not focus on a particular subspeciality, six reported on agents frequently associated with medication errors. Four studies reported that cardiovascular drugs were among the classes commonly associated with errors [43, 58, 59, 61]. Gastrointestinal drugs [49, 58], antimicrobials [43, 61], vitamins [44, 49], and analgesics [43, 49] were reported as the most common drug classes associated with errors in two studies each.

### Risk of bias

The overall quality of studies was assessed to be moderate (Electronic Supplementary 1, Table 2): five studies were at low risk of bias, thirteen were at moderate risk, and six were at high risk. The key limitations centered on potential biases with the recruitment and sampling procedures.

**Table 2:** Outcomes of studies reporting on contributory factors to ME

Author	Active failures and types	Latent conditions and types
Abramson E et al [43]	Mistake: prescribing errors Violation: inappropriate use of abbreviations	Lack of e-prescribing
Abramson E et al [44]	Mistake: wrong medication components Violation: inappropriate use of abbreviations	Performance deficit (wrong patient direction)
Bell S et al. [45]	NR	Misunderstanding and miscommunication
Bicket M et al. [46]	Violation: inappropriate use of abbreviations, incomplete prescriptions	Inadequate training/knowledge (physicians make less errors as compared to trainee and nurses) Lack of e-prescribing
Dempsey J et al. [47]	Mistake: prescribing errors	Inadequate training/knowledge Fragmentation of care
Howard M et al.[48]	NR	Inadequate training/knowledge (specially for specific population: female, elderly, altered kidney function)
Prasad D et al.[49]	Slips: dispensing errors (wrong quantity) Lapses: omission of diagnosis	Inadequate training/knowledge (specially for specific population: female) Heavy workload and lack of time Interruption and distraction in the environment Absence of quality assurance into academic education
Priya K et al. [50]	Mistake: allergic reaction	NR
Shakuntala B et al. [51]	NR	NR
Thakur H et al. [52]	NR	NR
Al-Khani S et al. [53]	Slips: look alike or sound alike, selecting the incorrect medication	Performance deficit (duplicate therapy)
Assiri G et al. [54]	NR	Inadequate training/knowledge (specially for specific population: elderly, polypharmacy, male)
Carollo J et al.[55]	Slips: dispensing errors (wrong medication) Lapses: omission of medication components Violation: inappropriate use of abbreviations	Lack of documentation (duplicate dose administered) Performance deficit Lack of e-prescribing Unstandardized prescription process
Duarte et al. [56]	Mistake: prescribing errors Slips: incorrect patient Violation: incomplete prescriptions	NR
Al Khawaldeh T et al. [57]	NR	Inadequate training/knowledge Performance deficit (not checking prescription and stability, lack of double checking) Heavy workload and lack of time Shortage of staff Lack of resources (protective equipment)
Belaiche S et al. [58]	NR	Inadequate training/knowledge (specially for specific population: multiple comorbidities and polypharmacy) Fragmentation of care Heavy workload and lack of time
Hernández S et al. [59]	Slip: dispensing errors	Inadequate training/knowledge
Kim G et al. [60]	Mistake: wrong dose	Inadequate training/knowledge
Lee P et al. [61]	NR	Inadequate training/knowledge (specially for immunosuppressant which have narrow therapeutic window) Performance deficit (duplicate therapy)
Niriayo Y et al.[62]	NR	Inadequate training/knowledge (specially for specific population: female, elderly, multiple concomitant comorbidities and polypharmacy, new guidelines and evidence) Performance deficit (duplicate therapy) Lack of patient involvement in decision making
Ojeh V et al. [63]	Mistake: allergic reaction Slips: incorrect patient	Inadequate training/knowledge (specific to HIV due to the changes in guidelines and complex nature of HIV) Performance deficit (duplicate therapy) Unstandardized prescription process
Rouhani M et al. [64]	Violation: noncompliance to protocol (standard form)	Inadequate training/knowledge (standard form and calculations)
Shaikh A et al. [65]	Violation: inappropriate use of abbreviations, incomplete prescriptions	Inadequate training/knowledge Lack of e-prescribing
Shrestha R et al. [66]	Mistake: prescribing errors Violation: incomplete prescriptions, carelessness, prescribing by brand name	Inadequate training/knowledge Performance deficit Lack of guidelines

*ME* Medication errors; *NR* Not reported

### Methods and resources used to identify and validate medication errors

Twenty studies (83.3%) provided descriptions, in various levels of details, about the approaches used to obtain prevalence data. Reviewing prescriptions/patients’ records was the predominant method [43–48, 50, 51, 54–56, 59, 60, 66]. Pharmacists were the professionals mostly performing these revisions, followed by nurses, physicians, and multidisciplinary teams. Other methods included pharmaceutical consultations [58, 61–63], direct observation [57], and reviewing medication errors reports [53]. Ten studies briefly described the instruments/standards used to identify medication errors [43–46, 54–56, 62, 65, 66]. Eight studies conducted validation of outcomes, for which double-checking or consensus were used [43, 47, 53, 54, 58, 59, 61, 62]. Only four studies had uniform training of the individuals involved in the identification and verification processes [43, 44, 62, 66].

### Prevalence of medication errors without associating them with the stages of the medication use process

The rate of overall medication errors was investigated in nine studies (Electronic Supplementary 1, Table 3), of which one study focused on “clinically important” medication errors [54] and another on “serious” medication errors as reported by patients [45]. The latter two studies did not provide a definition for clinically important and serious errors; however Assiri et al. (2019) reported that they adapted a previously published definition [54].

**Fig. 2:**
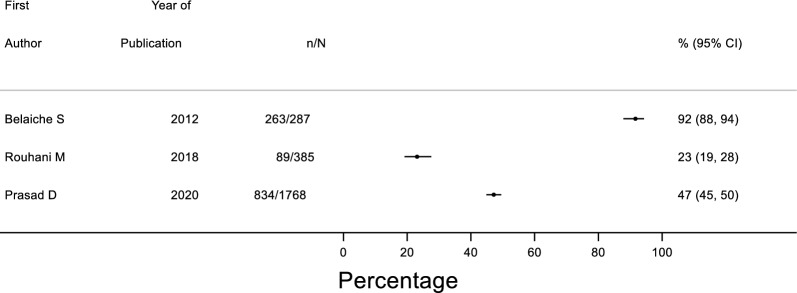
Forest plot of medications with errors as a proportion of total medications

The proportion of prescribed drugs associated with medication errors ranged between 23–92% in the three studies that used the total number of drugs as a reporting unit (Fig. 2). In the five studies that used the number of patients as a reporting unit, the rate of errors per patient ranged from 1.06 to 6.26 (Electronic Supplementary 2, Fig. 1). The rate of clinically important medication errors per patient was 0.08 in family medicine clinics [54], while patients attending general ambulatory practice reported 50 serious medication errors (14% of the overall observed errors) [45]. It is worth noting that the latter study evaluated patient-reported errors and did not solely focus on medication [45].

The proportion of prescriptions (could contain one or more drugs) with at least one medication error ranged between 42 and 56% in two studies [46, 49]. In a further study that focused on older adults, the incidence rate of medication errors was found to be 12.5 per 100 person-years (95% CI 9.4–16.2) [59].

### Prevalence of medication errors according to the medication-use process

Of the 24 studies, 19 (79.2%) reported prescribing errors, five administration errors, three dispensing errors, and one monitoring errors. Electronic Supplementary 1, Table 4 represents the outcomes of studies reporting prevalence data according to the medication use process and type of prescribing errors.

**Fig. 3:**
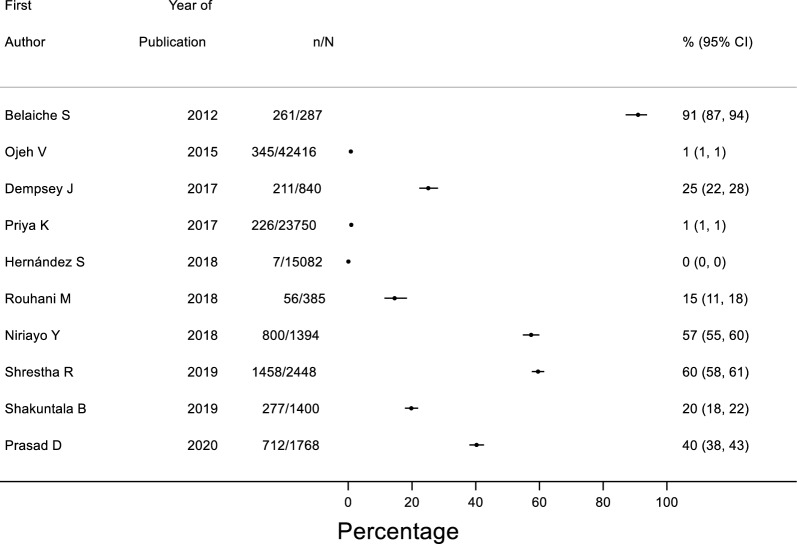
Forest plot of medications with prescribing errors as a proportion of total medications

A wide range of prevalence of prescribing errors was reported with errors ranging from 0–91% of all medications prescribed (Fig. 3), while the rate of prescribing errors per patient ranged between 0 and 6.21 in 13 studies (Electronic Supplementary, Fig. 2). Among studies that reported denominators other than patients and medications, 156 (7.8%) prescriptions were found to have clinically important prescribing error in 2000 patients attending family medicine clinics [54]. Another study focused on nonsteroidal anti-inflammatory drugs (NSAIDs) and reported 458 prescribing errors in 479 prescriptions [65]. Al-Khani et al. (2013) reported 2073 prescribing errors; however this study did not report a denominator [53].

Among studies reporting administration errors (*n* = 5), four used the total number of patients as a denominator, with the proportion of patients with errors ranging from 0% [59] to 39.2% [64]. One study had 654 administration processes, projecting 15,042 opportunities for error, of which 4112 (27.3%) errors were detected [57]. This study focused on intravenous chemotherapy and defined administration errors as any deviation from hospital protocol, which incorporated aseptic techniques [57].

Dispensing errors were assessed in three studies. The first study focused on chemotherapy and detected 21 (1.5%) dispensing errors in 1403 patients [55]. The second study recruited older patients, with an incident rate of 20.7 per 100 person-years [59] whilst the third, reported 122 (22.4%) errors in 544 patients [49].

Only one study reported monitoring errors, with six (0.3%) clinically important errors in a pool of 2000 patients [54].

### Prevalence based on the types of prescribing errors

Nineteen studies (79.2%) classified types of prescribing errors (Electronic Supplementary 1, Table 4), with wrong dose/strength (*n* = 16) being reported by the most studies, followed by wrong/suboptimal drug (*n* = 11), errors in relation to duration of use (*n* = 7), and errors in relation to frequency of prescribed medications (*n* = 7). Other types were wrong route, wrong/omitted patient directions, drug-drug interactions, contraindication, and others (e.g. duplicate therapy, inappropriate use of abbreviations).

**Fig. 4:**
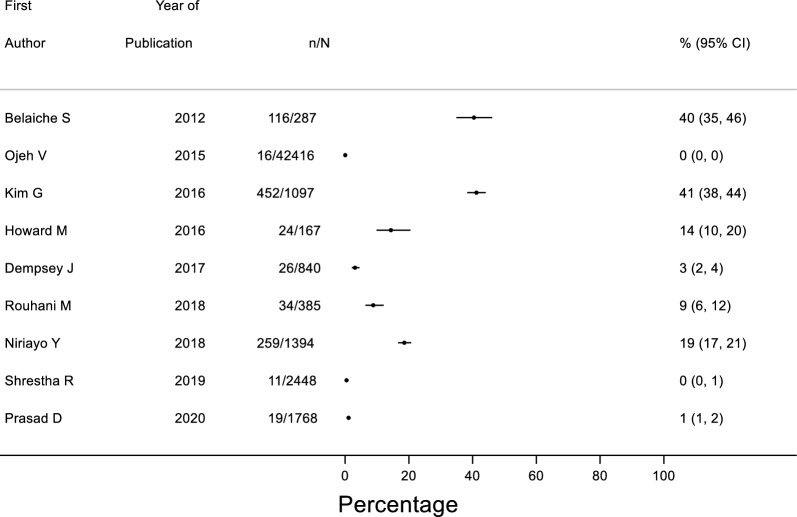
Forest plot of medications with dosing errors as a proportion of total medications

A wide range of prevalence of dosing errors (overdose or underdose) was reported with errors ranging from 0–41% of all medications prescribed (Fig. 4). Among studies (*n* = 13) that reported the total number of patients, the rate of dosing errors per patient ranged from 0 to 2.76 (Electronic Supplementary 2, Fig. 3). In a retrospective study that had prescriptions as a denominator, 112 (25.5%) dosing errors were detected in 479 prescriptions [65]. Another study conducted in ambulatory centers found 1099 dosing errors but no denominator was provided [53].

The range of prevalence of wrong or suboptimal drug errors per prescribed medications was found to range between 0 and 19% of all medications prescribed (Electronic Supplementary 2, Fig. 4), while nine studies found a range of dosing error rates of 0.01 to 0.79 per patient (Electronic Supplementary 2, Fig. 5). Al-Khani et al. (2013) reported 242 dosing errors but no denominator was reported [53].

Among studies assessing errors in relation to duration of use (n = 7; 29.2%), only one reported the total number of drugs, of which 14% (*n* = 196) had an error [51]. Four studies reported the total number of patients, with the rate per patient of wrong duration of use ranging from 0.0 to 0.38 (Electronic Supplementary 2, Fig. 6). In a retrospective study that focused on NSAIDs, 44 duration of use errors were identified in 479 prescriptions [65]. Around 2.4% of prescribing errors were found to be duration of use errors in another retrospective study that did not provide a denominator [53].

Seven studies (29.2%) reported errors in relation to frequency of prescribed medications, of which only two prospective studies conducted in hospital-based outpatient departments provided the total number of medications. The prevalence per prescribed medications was 5% in the study with a 4-month follow-up duration [51], while it was 0.0% in another study that had 12-month follow-up duration (Electronic Supplementary 2, Fig. 7). Four studies reported the total number of patients and the proportion of frequency errors per patient ranged from 0.01 to 0.08 (Electronic Supplementary 2, Fig. 8). In a study that reported the overall number of prescriptions and focused on NSAIDs, only nine (1.88%) frequency errors were detected in 479 prescriptions [65]. Around 8.7% of prescribing errors were found to be frequency errors in a study that did not report a denominator [53].

### Severity of medication errors

Out of the six studies (25%) that reported severity outcomes, four described the method used for categorization [50, 56, 59, 66]. Various methods were used to classify severity; hence we were unable to identify common patterns. The number of ranks (e.g. mild, moderate) in the used severity scales varied from two [49, 55] to seven ranks [59]. Although consequences from medication errors were mostly mild to moderate and were not linked to patient harm, potentially lethal incidents were reported in one study [56].

### Contributory factors to medication errors

Of the 24 studies, 22 (91.7%) reported the contributory factors leading to medication errors (Table 2). None of these studies used theories/models/frameworks during data collection or analysis. According to our synthesis using Reason’s model, 20 studies (90.9%) reported that latent conditions contributed to medication errors, while 15 studies (68.2%) reported active failures.

Inadequate training or knowledge was a common latent condition reported by studies (Table 2). Examples included poor training specific to special populations (particularly older patients with polypharmacy) and lack of knowledge related to updated therapeutic guidelines. Performance deficits were also common, largely due to duplicate therapy.

Among studies that reported active failures, eight highlighted mistakes, eight highlighted violations, six highlighted slips, and two highlighted lapses (Table 2). Inappropriate use of abbreviations and incomplete prescriptions were example of violations. There was considerable diversity among the contributory factors leading to mistakes, with examples including dosing errors due to failure to account for risk factors (e.g. elevated creatinine) and prescribing a medication in a patient with known allergy.

### Intervention to mitigate medication errors in outpatient and ambulatory settings

Only two types of interventions were identified from the seven studies that implemented interventions to minimize medication errors. Pharmacist-delivered interventions [47, 50, 56, 58, 61, 63] were the most commonly evaluated, while only one study evaluated the effectiveness of e-prescribing software [43].

Among pharmacist-led interventions, three studies conducted direct consultation sessions with patients [58, 61, 63], two performed revisions of electronic records/prescriptions [50, 56], and one combined records checking with medication reconciliation [47]. Four out of the six studies also explored intervention subtypes (e.g. change to alternative medication, adjust dose) [47, 56, 58, 63]; however only one reported the methodology used for this categorization [56]. The most common types of interventions related to adjustments of one or more regimen components (e.g. dose, duration), switch to alternative therapy, adding medications, and stopping unnecessary medications.

The study that implemented e-prescribing was the only one to report the number of errors before and after the intervention [43]. The remainder reported the total number of interventions or preventable overall medication errors. The e-prescribing study analyzed 9385 prescriptions for 5955 patients and assessed that 19,571 out of 19,956 errors could have been prevented by implementing the basic and advanced versions of the clinical decision support (CDS) systems [43]. All illegibility errors resolved after applying the basic version of the system [43]. Among studies that did not report the counts pre and post intervention, the number of interventions ranged from 64 in a study that included 60 patients [47] to 843 in a population of 1271 patients [61].

## Discussion

### Statement of key findings

The findings from this systematic review highlighted that medication errors were common (prevalence of 23–92% for prescribed drugs) in outpatient and ambulatory settings, while acknowledging variation in the ranges of prevalence estimates in individual studies. Prescribing errors were the most frequently studied type of errors, with a prevalence of 0–91% errors for prescribed medication. The most common incident types were dosing errors (prevalence of 0–41% for prescribed drugs) and suboptimal/wrong drug errors (prevalence of 0–19% for prescribed drugs), followed by errors in relation to duration of use and frequency of prescribed medications. Notably, latent conditions, including inadequate training or knowledge, were more common than active failures. Among active failures, mistakes and violations were the most frequent contributory factors. Pharmacist-led interventions and e-prescribing software have been studied to reduce medication errors in these settings; however studies lacked randomized design and long-term follow-up.

### Strengths and weaknesses

This is the first systemic review of studies exploring medication errors in outpatient and ambulatory settings. A theoretical approach to classifying contributory factors was adopted, which enhances the validity of our outcomes and facilitates the development of interventions. Moreover, the included studies were conducted in different countries with various clinical practices and healthcare systems; and there was no restriction on the medical subspeciality which could increase the generalizability and transferability of our findings.

The current review was limited to the literature published in English language. The synthesis of contributory factors was subjected to reporting bias as it relies on what has been reported by the original studies. Classifying contributory factors according to Reason’s model could be subject to interpretation bias, particularly when the error circumstances and conditions are not thoroughly presented. Additionally, evidence suggests that medication errors are underreported [67, 68]; hence studies that used incident reporting systems to quantify errors are likely to underestimate the true prevalence causing downward bias in the error rates in our review. Lastly, although meta-analysis on prevalence of medication errors was planned; it was not conducted due to the substantial between-studies heterogeneity. This could be due to multiple factors such as the variation in patient population, service specialty, length of follow-up, and the diverse definitions of medication errors adopted in included studies amongst others.

### Interpretation of findings

This review suggests that medication errors are common in outpatient and ambulatory settings. The range of prescribing errors rates from our findings was substantially higher than the rate of errors reported in a systematic review of 63 studies focusing on hospitalized patients [27]. While it is expected that medical problems and interventions in outpatient and ambulatory settings are less complex than in inpatient setting, these high numbers in the former settings necessitate attention from decision makers and other stakeholders to develop and implement prevention strategies. This high prevalence could be due to multiple factors such as the comparatively higher healthcare encounters occurring in outpatient and ambulatory settings, the tendency to report errors, or the less attention provided by policy makers to these settings [24, 25, 69]. Additionally, few studies reported zero errors identified in regards to certain medication sub-classes such as suboptimal/wrong drug errors. This could be attributed to method of identifying, validating and classifying errors, small sample size, and the short duration of the study amongst others. This high variation in the prevalence data could be due to the variation in clinical practices and healthcare systems in the countries where the included studies took place.

In line with previous research conducted in various populations and settings, prescribing errors were the most frequently studied, with dosing errors constantly being the most prevalent [29, 70, 71]. Whilst previous studies have reported active failures as the predominant contributory factors to medication errors [28, 70, 72, 73], latent conditions particularly the lack of knowledge and training were the most frequent in outpatient and ambulatory settings. The issue of supervisory and managerial inadequacies was also raised in studies that investigated the factors contributing to diagnostic errors in these settings [74–76].

Amongst active failures, mistakes (e.g. dosing errors due to failure to consider risk factors) and violations (e.g. inappropriate use of abbreviation) were the two most common factors. This finding is also distinct from what has been observed in other settings, in which slips, lapses, and mistakes were the three most common factors [28, 70, 72, 73]. It is worth pointing out that most violation cases in our review were attributed to the inappropriate use of medical abbreviation.

It is noteworthy that method of identifying and validating medication errors were poorly reported across studies. This reinforces findings from previous research that described the process of identifying medication errors as fraught with inaccuracies and systematic bias [28, 77]. Variations in the definition of medication error (and subclasses) were also noted amongst included studies. This emphasizes findings from previous studies suggesting inconsistencies in patient safety terminologies [31, 70, 78–80]. Additionally, all studies had cross-sectional or observational design with the lack of dissemination and implementation design such as randomized controlled trials. Moreover, most studies lacked a comprehensive description of the intervention characteristics (e.g. mode of contact, frequency of contact, setting where recipient received treatment, service provider actions) and outcomes. Therefore, no conclusions could be drawn about the effectiveness of the proposed interventions.

There was notable variation regarding the classes of medications associated with errors; however in line with previous systematic reviews, cardiovascular drugs were the most frequently reported therapeutic group [28, 30, 81]. Some treatment modalities that are not usually seen in other settings have emerged in our review such as analgesics and vitamins [30, 71]. These classes might seem simple as they mainly treat mild conditions. Nonetheless, some of them have many restrictions and could lead to serious adverse events such as NSAIDs and opioids [82].

### Implications for practice and research

Medication errors are common in outpatient and ambulatory settings even though there was variation in the data. This finding highlights the need to reduce medication errors in these settings. Our comprehensive synthesis of contributory factors facilitates the development of multifaceted theory-based interventions tailored to the factors identified in this review. Theory-based interventions are expected to yield promising outcomes as other methods of developing interventions (e.g. pragmatic approach) have been proven ineffective [83–88].

Latent conditions were the main contributory factors in this review hence it is believed that dedicating more efforts and allocating more resources by policy makers, managers, and other stakeholders towards these settings will have a positive impact. The review also emphasizes the insufficient knowledge and training amongst healthcare professionals therefore educational sessions that are based on structured needs assessment are expected to mitigate medication errors in these settings [5, 89]. Furthermore, most errors occurred at the prescribing stage. Previous studies showed that pharmacist-led and technology-facilitated interventions lead to a reduction in prescribing errors and improvement in health outcomes [8, 90, 91], hence they could also be beneficial in these settings.

Future research should focus on the development of theory-based multifactorial interventions that incorporate managers, pharmacists, technologies, and education. The UK Medical Research Council (MRC) framework could be used to develop effective complex interventions [92]. This framework incorporates theory to identify behavioral determinants to target in subsequent interventions and to ensure proper translation into practice [92]. Moreover, studies with high quality design (i.e. randomized controlled trials) that aim to evaluate the long-term outcomes of interventions are needed to accurately measure the effectiveness of these interventions.

Poor reporting of the method of identifying and validating medication errors was recognized across studies. It is strongly encouraged that future researchers adopt a well-established and validated methodology to identify medication errors and to train individuals involved in the process. It also is recommended to address issues related to validation of identified errors, which could be conducted through multiple methods such as double-checking and calculating interrater reliability. Additionally, evidence suggests that up to 60% of medication errors are under-reported, which is largely attributed to the lack or inefficient incident reporting systems in healthcare organizations [67, 93, 94]. The use of efficient and effective incident reporting system is key to improving patient safety and care quality. In addition, healthcare accreditation movement (such as the Joint Commission International (JCI)) that is being increasingly adopted by healthcare organizations across the globe have the potential to reduce medical errors, improve performance and collectively enhance patient safety [95, 96].

Our findings also suggested inconsistencies in patient safety terminologies by the included studies, thus we recommend maintaining consistency in the terms used across each study and to provide definitions for each term. This is of particular importance, as variation might lead to confusion regarding the phenomenon of interest which could affect the reliability of the outcomes.

None of the included studies followed a structured approach to classify contributory factors. Adopting a theory-based methodology such as Reason’s model will ensure that the identified contributory factors are inclusive and hence reduce the risk of reporting bias [31]. It also will increase our understanding of these factors which will facilitate the process of translating them into effective interventions.

## Conclusion

This systematic review suggests that medication errors in outpatient and ambulatory settings are highly prevalent; however wide variation in the prevalence range was observed across studies. The factors contributing to medication errors were mainly latent conditions, including the inadequate training or knowledge of healthcare practitioners in relation to special populations and updated therapeutic approaches. There is a need for the development of theory-based multifactorial interventions to minimize medication errors in outpatient and ambulatory settings. These interventions should include organizational and system-level strategies (e.g. effective resource allocation), multidisciplinary collaborations, effective integration of pharmacists, health information technology, as well as educational and training programs. Such approach enhances patient clinical, humanistic, and economic outcomes and subsequently improve the quality of care provided to patients. Randomized controlled trials are needed to develop and evaluate the long-term outcomes of complex interventions in these settings.

### Supplementary Information

Below is the link to the electronic supplementary material.

Supplementary file1 (DOCX 79 KB)
