# Supplementary material for: Prevalence, contributing factors, and interventions to reduce medication errors in outpatient and ambulatory settings: a systematic review
Source: Int J Clin Pharm. 2023 Sep 8;45(6):1359–77. doi: 10.1007/s11096-023-01626-5 (PMC10682158; doi:10.1007/s11096-023-01626-5)
Supplement: Supplementary file 1 — Supplementary file1 (DOCX 79 KB) [file 11096_2023_1626_MOESM1_ESM.docx]

**Supplementary 1: Tables**

| **Table 1. Articles excluded at the full text screening stage** | | |
| --- | --- | --- |
| **Number** | **Title of the excluded review** | **Reason for exclusion** |
| **1** | A cross-sectional study on prescribing and dispensing errors at a corporate hospital in South India | No isolation of adult outcomes |
| **2** | A prospective observational study of medication errors in general medicine department in a tertiary care hospital | Not focused on outpatient and ambulatory settings |
| **3** | Adverse events in psychiatry: A national cohort study in Sweden with a unique psychiatric trigger tool | Not focused on outpatient and ambulatory settings |
| **4** | An Internal Quality Improvement Collaborative Significantly Reduces Hospital-Wide Medication Error Related Adverse Drug Events | Not focused on outpatient and ambulatory settings |
| **5** | An observational study to evaluate the factors which influence the dispensing errors in the hospital pharmacy of a tertiary care hospital | Not focused on outpatient and ambulatory settings |
| **6** | Appropriateness of prescribing in selected healthcare facilities in Papua New Guinea | Not focused on medication errors |
| **7** | Clinical evaluation of pharmacists' interventions on multidisciplinary lung transplant outpatients' management: results of a 7-year observational study | No isolation of adult outcomes |
| **8** | Description of the role of pharmacist independent double checks during cognitive order verification of outpatient parenteral anti-cancer therapy | Not focused on medication errors |
| **9** | Descriptive analysis of medication errors reported to the Egyptian national online reporting system during six months | No isolation of adult outcomes |
| **10** | Drug errors and related interventions reported by united states clinical pharmacists: The american college of clinical pharmacy practice-based research network medication error detection, amelioration and prevention study | Not focused on outpatient and ambulatory settings |
| **11** | Epidemiology of adverse events and medical errors in the care of cardiology patients | Not focused on medication errors |
| **12** | Errors associated with outpatient computerized prescribing systems | Not focused on outpatient and ambulatory settings |
| **13** | Evaluation of faculty and non-faculty physicians' medication errors in outpatients' prescriptions in Shiraz, Iran | No isolation of adult outcomes |
| **14** | Frequency of ambulatory care adverse events in Latin American countries: the AMBEAS/PAHO cohort study | Not focused on medication errors |
| **15** | Frequency of and risk factors for medication errors by pharmacists during order verification in a tertiary care medical center | Not focused on outpatient and ambulatory settings |
| **16** | Frequency of medication errors in primary care patients with polypharmacy | Not focused on medication errors |
| **17** | Identification of medication errors through a monitoring and minimization program in outpatients in Colombia, 2018-2019 | Not English |
| **18** | Impact of clinical pharmacy services on medication errors in a multispecialty hospital | Not focused on outpatient and ambulatory settings |
| **19** | Impacts of Pharmacists-Managed Oncology Outpatient Clinic on Resolving Drug-Related Problems in Ambulatory Neoplasm Patients: A Prospective Study in China | Not focused on medication errors |
| **20** | Medication errors and adverse drug reactions in psychiatry department: A prospective observational study | No full text |
| **21** | Medication errors in a health care facility in southern Saudi Arabia | Not focused on outpatient and ambulatory settings |
| **22** | Medication errors in Auvergne-Rhone-Alpes: A prospective pilot study led in collaboration by regional vigilance and support structures | Not English |
| **23** | Medication errors in outpatient care in Colombia, 2005-2013 | Not focused on outpatient and ambulatory settings |
| **24** | Medication errors reported to the National Medication Error Reporting System in Malaysia: a 4-year retrospective review (2009 to 2012) | Not focused on outpatient and ambulatory settings |
| **25** | Medication incidents associated with outpatient computerized prescribing systems | No isolation of adult outcomes |
| **26** | Minimising prescription errors-a quality improvement project in the ophthalmology department in a tertiary referral hospital | Not focused on outpatient and ambulatory settings |
| **27** | Pharmacist's role in improving medication safety for patients in an allogeneic hematopoietic cell transplant ambulatory clinic | Not focused on outpatient and ambulatory settings |
| **28** | Potential risks in drug prescriptions to elderly: A cross-sectional study in the public primary health care system of Ourinhos micro-region, Brazil. | No full text |
| **29** | Prescription, Transcription and Administration Errors in Out- Patient Day Care Unit of a Regional Cancer Centre in South India | Not focused on outpatient and ambulatory settings |
| **30** | Prevalence of medication-related problems among patients with renal compromise in an Indian hospital | Not focused on outpatient and ambulatory settings |
| **31** | Prevalence, nature and potential preventability of adverse drug events - A population-based medical record study of 4970 adults | Not focused on outpatient and ambulatory settings |
| **32** | Prevention of medication errors in drug dispensation to outpatients, Colombia june 2014 to june 2015 | Not English |
| **33** | Reducing Medical Errors in Primary Care Using a Pragmatic Complex Intervention | Not focused on medication errors |
| **34** | Strategies to Reduce Medication Errors in Ambulatory Practice | Not focused on medication errors |
| **35** | The impact of electronic prescription on reducing medication errors in an Egyptian outpatient clinic | No isolation of adult outcomes |
| **36** | Update on Drug-Related Problems in the Elderly | Not focused on medication errors |
| **37** | What Safety Events Are Reported For Ambulatory Care? Analysis of Incident Reports from a Patient Safety Organization | Not focused on medication errors |

| **Table 2. Quality assessment of included studies** | | | | | | | | | | |
| --- | --- | --- | --- | --- | --- | --- | --- | --- | --- | --- |
| **Author, year of publication** | **Was the target population a close representation of the national population?** | **Was the sampling frame a true or close representation of the target population?** | **Was some form of random selection used to select the sample, OR, was a census undertaken?** | **Was the likelihood of non-response bias minimal?** | **Were data collected directly from the subjects (OR proxy)?** | **Was an acceptable case definition used?** | **Was the instrument that measured the parameter of interest shown to have reliability and validity (if necessary)?** | **Was the same mode of data collection used for all subjects?** | **Were the numerator(s) and denominator(s) for the parameter of interest appropriate** | **Overall risk of bias** |
| **Abramson E, 2011** | No | No | Yes | No | Yes | Yes | Yes | Yes | Yes | 3 (low risk) |
| **Abramson E, 2013** | No | No | No | No | Yes | Yes | Yes | Yes | Yes | 4 (moderate risk) |
| **Al Khawaldeh T, 2017** | No | No | No | Yes | Yes | Yes | Yes | No | Yes | 4 (moderate risk) |
| **Al-Khani S, 2013** | No | Yes | Yes | Yes | No | Yes | No | No | No | 5 (moderate risk) |
| **Assiri G, 2019** | No | Yes | Yes | No | No | Yes | Yes | Yes | No | 3 (low risk) |
| **Belaiche S, 2012** | No | Yes | No | No | Yes | No | No | No | No | 3 (low risk) |
| **Bell S, 2020** | Yes | Yes | No | No | Yes | No | Yes | Yes | Yes | 6 (moderate risk) |
| **Bicket M, 2018** | Yes | No | No | Yes | Yes | No | Yes | Yes | Yes | 3 (low risk) |
| **Carollo J, 2017** | No | Yes | No | No | Yes | No | No | Yes | No | 4 (moderate risk) |
| **Dempsey J, 2017** | Yes | Yes | No | No | Yes | Yes | Yes | Yes | No | 5 (moderate risk) |
| **Duarte N, 2018** | No | No | No | No | Yes | Yes | No | Yes | Yes | 4 (moderate risk) |
| **Hernández S, 2018** | No | Yes | No | Yes | Yes | No | No | Yes | No | 4 (moderate risk) |
| **Howard M, 2016** | No | No | No | No | Yes | Yes | Yes | Yes | Yes | 4 (moderate risk) |
| **Kim G, 2016** | No | Yes | No | No | No | Yes | Yes | Yes | Yes | 2 (low risk) |
| **Lee P, 2016** | No | No | No | No | Yes | Yes | Yes | Yes | Yes | 5 (moderate risk) |
| **Niriayo Y, 2018** | No | Yes | No | Yes | Yes | Yes | Yes | Yes | Yes | 5 (moderate risk) |
| **Ojeh V, 2015** | No | No | No | No | Yes | Yes | No | Yes | Yes | 7 (high risk) |
| **Prasad D, 2020** | No | No | Yes | No | Yes | No | No | Yes | Yes | 7 (high risk) |
| **Priya K, 2017** | No | No | No | Yes | Yes | No | No | No | No | 7 (high risk) |
| **Rouhani M, 2018** | No | No | No | No | Yes | No | No | Yes | Yes | 7 (high risk) |
| **Shaikh A, 2017** | No | No | No | No | Yes | No | No | Yes | No | 4 (moderate risk) |
| **Shakuntala B, 2019** | No | No | No | No | Yes | No | No | No | Yes | 9 (high risk) |
| **Shrestha R, 2019** | No | Yes | Yes | Yes | Yes | No | No | Yes | No | 3 (low risk) |
| **Thakur H, 2013** | No | No | No | No | No | No | No | No | No | 6 (moderate risk) |
| Low risk of bias: final score 0-3 points; moderate risk of bias: final score 4-6; high risk of bias: final score 7-9 | | | | | | | | | | |

| **Table 3. Outcomes of studies reporting on the prevalence of overall medication errors (n=9)** | | | | |
| --- | --- | --- | --- | --- |
| **Author, year of publication** | **Method specific to prevalence data** | **Duration of prevalence data collection** | **Total number of observations (denominator)** | **Number of overall ME** |
| **Assiri G, 2019 (43)** | In-depth electronic record screening was conducted. Clinically important errors as defined by the PINCER trial were identified. A second trained reviewer undertook the independent assessment of a random 10% of the sample of records. Any discrepancy was discussed and resolved through double-checking of records or arbitration if a decision could not be reached | 15 months | 2,000 patients | 162 patients with clinically important errors |
| **Belaiche S, 2012 (47)** | Pharmaceutical consultations by the pharmaceutical team (1 senior clinical pharmacist and 1 clinical pharmacy resident) | 15 months | 42 patients (350 pharmaceutical consultations, 287 drugs) | 263 ME |
| **Bell S, 2020 (34)** | To help focus patients on their notes, the adapted survey included a screenshot of the location of notes on patient portal. The survey included 4 questions about mistakes. Included Likert scale, yes/no, multiple choice questions, and open-ended questions | 5 months | 22,889 patients | 50 serious ME |
| **Bicket M, 2018 (35)** | One investigator examined prescriptions for errors according to three standards: 1) PE based on “best practice” guidelines; 2) The Joint Commission recommendation for two patient identifiers; and 3) the DEA Practitioner’s Manual Valid Prescription Requirements. A second investigator independently examined a subset of handwritten prescriptions and prescriptions noted to contain at least one error for confirmation. Any discrepancies were resolved by consensus | 15 days | 451 patients (510 prescriptions) | 214 prescriptions contained at least one error |
| **Carollo J, 2017 (44)** | Data was collected using an instrument divided into four sections by evaluating patients’ medical records who received care, technical complaints forms and incident notifications | 3 months | 1,403 patients (1,403 healthcare procedures) | 4867 ME |
| **Hernández S, 2018 (48)** | The necessary data to evaluate the incidence of ME were obtained from reports, medical records review, and pharmacy dispensing medication profiles. A pharmacy student performed the data collection and records review under supervision of a clinical pharmacy preceptor and the pharmacy department director, both Doctors of Pharmacy | 4 years | 2,218 patients | 93 ME |
| **Prasad D, 2020 (38)** | NR | 6 months | 544 patients (544 prescriptions,  1768 drugs) | 834 ME |
| **Rouhani M, 2018 (53)** | NR | 6 months | 84 patients (217 cycles, 385 drugs) | 89 ME |
| **Thakur H, 2013 (41)** | NR | 5 months | 100 patients | 171 ME |
| **ME: medication errors; NR: not reported** | | | | |

| **Table 4. Outcomes of studies reporting prevalence data according to the medication use process and type of prescribing errors** | | | | | | |
| --- | --- | --- | --- | --- | --- | --- |
| **Author, year of publication** | **Methods specific to prevalence data reported according to the use process** | **Methods specific to classifying ME according to the type of prescribing error** | **Duration of prevalence data collection** | **Total number of observations (denominator)** | **Number of errors according to the medication use process** | **Number of errors according to the type of prescribing errors** |
| **Abramson E, 2011 (32)** | A physician trained a nurse and pharmacist in an identical manner using well-used, standardized methodology. Interrater reliability was determined by having the pharmacist and nurse evaluate the same random sample of 2% of the data and calculating the k score | Errors classified in accordance with definitions from the Institute of Medicine | 2 weeks | 5955 patients (9385 prescriptions) | -PE: 19,956 | -Wrong dose/strength: 736  -Wrong frequency: 192  -Wrong duration: 333  -Wrong/omitted patient direction: 394  -Others: 18,301 |
| **Abramson E, 2013 (33)** | An experienced nurse reviewer evaluated all prescriptions. This nurse had previously been trained to apply extensively used and standardized methodology that includes error classification and identification | Errors classified in accordance with definitions from the Institute of Medicine | 2 weeks | 920 patients (1905 prescriptions) | -PE: 71 | -Wrong dose/strength: 8  -Wrong frequency: 17  -Wrong duration: 5  -Wrong/omitted patient direction: 7  -Wrong route: 1  -Others: 32 |
| **Al Khawaldeh T, 2017 (46)** | Clinical pharmacist undertook direct observation of 5 outpatient nurses who administered chemotherapy drugs | Not studied | 6 weeks | 334 drugs administered/ prescriptions | -Administration errors: 965 | Not studied |
| **Al-Khani S, 2013 (42)** | All PE reported to the electronic reporting system (voluntary) by the pharmacist and reviewed by the quality control department and medication safety officer. Reports were thoroughly reviewed and evaluated by two researchers | Errors classified in accordance with the classification of the electronic reporting system | 21 months | NR | -PE: 2073 | -Wrong dose/strength: 1099  -Wrong/suboptimal drug: 242  -Wrong frequency: 180  -Wrong duration: 49  -Wrong route: 30  -Other: 473 |
| **Assiri G, 2019 (43)** | In-depth electronic record screening was conducted. Clinically important errors as defined by the PINCER trial were identified. A second trained reviewer undertook the independent assessment of a random 10% of the sample of records. Any discrepancy was discussed and resolved through double-checking of records or arbitration if a decision could not be reached | NR | 15 months | 2,000 patients | -Clinically important PE: 156  -Clinically important monitoring errors: 6 | -Wrong/suboptimal drug: 33  -DDI: 2  -Combination of errors: 34  Other: 87 |
| **Belaiche S, 2012 (47)** | Pharmaceutical consultations by the pharmaceutical team (1 senior clinical pharmacist and 1 clinical pharmacy resident) | NR | 15 months | 42 patients (350 pharmaceutical consultations, 287 drugs) | -PE: 261  -Administration errors: 2 | -Wrong dose/strength: 116  -Wrong/suboptimal drug: 19  Others: 126 |
| **Carollo J, 2017 (44)** | Data was collected using an instrument divided into four sections by evaluating patients’ medical records who received care, technical complaints forms, and incident notifications | NR | 3 months | 1,403 patients (1,403 healthcare procedures) | -PE: 4819  -Dispensing errors: 21  -Administration errors: 27 | -Wrong dose/strength: 457  -Wrong/suboptimal drug: 480  -Wrong duration: 529  -Wrong rout: 21,  -Others: 3,332 |
| **Dempsey J, 2017 (36)** | A pharmacist reviewed each patient’s medical profile and medication list to identify and categorize ME. Each documented ME was verified by a second pharmacist | Errors classified in accordance with the drug-related problems described by Hepler and Strand | 5 months | 60 patients | -PE: 211 | -Wrong dose/strength: 26  -Wrong/suboptimal drug: 46  -DDI: 90  -Contraindication: 11  -Others: 38 |
| **Duarte et al, 2018 (45)** | A pharmacist evaluated prescriptions according to a set criteria such as using treatment protocol and dosage calculations | NR | 6 months | 780 patients (3526 prescriptions) | -PE: 220 | -Wrong dose/strength: 79  -Wrong/suboptimal drug: 58  -Wrong frequency: 4  -Wrong duration: 2  -Others: 77 |
| **Hernández S, 2018 (48)** | The necessary data to evaluate the incidence of ME were obtained from reports, medical records review, and pharmacy dispensing medication profiles | Not studied | 4 years | 2,218 patients | -PE: 7  Dispensing errors: 86  -Administration errors: 0 | Not studied |
| **Howard M, 2016 (37)** | Not studied | Appropriateness of dose (FDA approved doses) was determined at the time of data collection | 6 months | 167 patients (167 drugs/ prescriptions) | Not studied | -Wrong dose/strength: 24 |
| **Kim G, 2016 (49)** | Not studied | Clinical pharmacists reviewed prescriptions and evaluated the adherence to renal dosing according to Micromedex/Lexicomp | 40 days | 828 patients (1097 drugs) | Not studied | -Wrong dose/strength: 452 |
| **Lee P, 2016 (50)** | At each visit, the patient was seen by the transplant pharmacist after consultation with the physician. The pharmacist reviewed and optimized the medication regimens. Any ME or discrepancies identified during the consultation were discussed with the physicians-in-charge | Errors classified according to Strand criteria and American College of Clinical Pharmacy guidelines for therapeutic interchange | 19 months | 1271 patients (3581 prescriptions) | -PE errors: 843 | -Wrong dose/strength: 254  -Wrong/suboptimal drug: 75  -DDI: 3  -Others: 511 |
| **Niriayo Y, 2018 (51)** | Patients were interviewed consecutively using the interview questionnaire and their respective medical chart was retrieved using the retrieval checklist. Clinical pharmacists, nurses and physician were involved in data collection. Training and orientation were given. ME were identified using the Cipolle’s method followed by a consensus meeting with a panel of experts. The experts further refined ME identification method setting based on treatment and literature reviews | Errors classified in accordance with the Cipolle’s method followed by a consensus meeting with a panel of experts. The experts further refined ME classification method based on guidelines and literature reviews | 12 months | 340 patients (1389 drugs) | -PE: 800 | -Wrong dose/strength: 259  -Wrong/suboptimal drug: 267  -Others: 274 |
| **Ojeh V, 2015 (52)** | At every fill/refill visit, pharmacists engaged in face-to-face interaction with the patient to verify the accuracy of prescriptions with consideration to clinical and laboratory parameters | NR | 8 months | 9,339 patients (42,416 prescriptions) | -PE: 345 | -Wrong dose/strength: 16  -Wrong/suboptimal drug: 110  -DDI: 6  -Contraindication: 2  -Others: 211 |
| **Prasad D, 2020 (38)** | NR | NR | 6 months | 544 patients (544 prescriptions,  1768 drugs) | -PE: 712  -Dispensing error: 122 | -Wrong dose/strength: 19  -Wrong/suboptimal drug: 75  -Others: 618 |
| **Priya K, 2017 (39)** | Pharmacists auditing e-prescriptions | PE classified according to NCCMERP. In addition to pharmacist professional knowledge, other clinical guidelines | 12 months | 23,750 drugs | -PE: 226 | -Wrong frequency: 78  -DDI: 56  -Other: 6 |
| **Rouhani M, 2018 (53)** | NR | NR | 6 months | 84 patients (217 cycles, 385 drugs) | -PE: 56  -Administration errors: 33 | -Wrong dose/strength: 34  -Others: 22 |
| **Shaikh A, 2017 (54)** | Identifying errors as per the WHO prescription writing guidelines, authenticated drug references drug information book and the British National Formulary (BNF) | NR | NR | 479 prescriptions | -PE: 458 | -Wrong dose/strength: 112  -Wrong frequency: 9  -Wrong duration: 44  -Wrong route: 89  -DDI: 92  -Others: 112 |
| **Shakuntala B, 2019 (40)** | The required information recorded prospectively in a specially designed form (case record) from the outpatient department prescription letter of every patient in the study | NR | 4 months | 900 patients (900 prescriptions, 1400 antibiotic) | -PE: 277 | -Wrong frequency: 70  -Wrong duration: 196  -Wrong route: 11 |
| **Shrestha R, 2019 (55)** | The trained pharmacy personnel collected data on the WHO prescribing indicators and PE (parameters were prepared by studying WHO practical manual and previous studies) retrospectively | NR | 2 months | 770 prescriptions, 2448 drugs | -PE: 1458 | -Wrong dose/strength: 11  -Wrong/suboptimal drug: 4  -Wrong route: 5  -DDI: 249  -Others: 1189 |
| **ME: medication errors; PE: prescribing errors; NR: not reported; DDI: drug-drug interactions** | | | | | | |

**Supplementary 2: Figures**

**Fig 1.** Forest plot of the rate of overall medication errors per patient

**Fig 2.** Forest plot of the rate of prescribing errors per patient

**Fig 3.** Forest plot of the rate of dosing errors per patient

**Fig 4.** Forest plot of medications with wrong/suboptimal drug errors as a proportion of total medications

**Fig 5.** Forest plot of the rate of wrong/suboptimal errors per patient

**Fig 6.** Forest plot of the rate per patient of duration of use errors

**Fig 7.** Forest plot of medications with frequency errors as a proportion of total medications

**Fig 8.** Forest plot of the rate per patient of frequency of prescribed medication errors
